# Landelijke diabetesregistratie DPARD: vijf jaar succesvol op weg

**DOI:** 10.1007/s12467-023-1212-9

**Published:** 2023-03-08

**Authors:** Silvia A. G. de Vries, Jessica C. G. Bak, Erik H. Serné, Mark H. H. Kramer, Carianne L. Verheugt

**Affiliations:** 12467170754001 Amsterdam Universitair Medische Centra, Amsterdam, Nederland; 12467170754002 Afdeling Vasculaire Geneeskunde, Amsterdam Universitair Medische Centra, Meibergdreef 9, 1105 AZ Amsterdam, Nederland

## Achtergrond DPARD

In 2017 werd op Wereld Diabetes Dag DPARD (Dutch Pediatric and Adult Registry of Diabetes) opgericht, de nationale registratie voor diabetespatiënten behandeld in de tweede en derde lijn.^1^ In DPARD worden zowel kinderen als volwassenen geregistreerd over langere tijd. In Nederland worden naar schatting 185.000 patiënten met diabetes mellitus behandeld in de tweede of de derde lijn,^2^ een aantal dat naar verwachting toe zal nemen als gevolg van overgewicht en vergrijzing. Het exacte aantal polikinisch behandelde diabetespatiënten is echter onbekend. Ook is weinig bekend over de kenmerken en vooruitzichten van deze patiënten. Om de kwaliteit van diabeteszorg in de toekomst te garanderen, is meer kennis nodig over deze groep diabetespatiënten. Het primaire doel van DPARD is om op lokaal, regionaal en landelijk niveau beter inzicht te krijgen in alle aspecten van de chronische poliklinische diabeteszorg, teneinde de kwaliteit van diabeteszorg op elk van deze niveaus verder te verbeteren. 

DPARD is opgericht door Stichting BIDON (Basisstructuur Innovatief Diabetes Onderzoek Nederland), een landelijk consortium van internisten, kinderartsen en diabetespatiënten. Sinds 2018 valt DPARD onder de vleugels van DICA, een stichting die 22 landelijke kwaliteitsregistraties beheert, met als doel de kwaliteit van zorg inzichtelijk te maken. Het bestuur van DPARD is de Clinical Audit Board (CAB), bestaande uit de bestuursleden van Stichting BIDON. In de wetenschappelijke commissie nemen afgevaardigden deel van centra die deelnemen aan DPARD. De CAB is in samenspraak met de wetenschappelijke commissie verantwoordelijk voor het vaststellen van de jaarlijks aan te leveren landelijke kwaliteitsinformatie, de doorontwikkeling van de registratie en bewaking van de datakwaliteit.

In DPARD worden gegevens geregistreerd die betrekking hebben op patiëntkarateristieken, diagnostiek, behandeling, uitkomsten en complicaties. Alle typen diabetes mellitus worden geïncludeerd, behoudens zwangerschapsdiabetes, aangezien dit in principe een tijdelijke aandoening is. Data worden direct vanuit het elektronisch patiëntendossier (EPD) verzameld. Inclusie vindt hierbij plaats op basis van DBC- (diagnose-behandelcombinatie)declaratiecodes. Diabetesdiagnose, classificatie, complicaties en comorbiditeiten worden vastgelegd met ICD-10-codes, wat aansluit bij de manier waarop gegevens op landelijk niveau in Nederland in elk EPD vastgelegd worden. Data vanuit het EPD wordt gebundeld in een groot databestand. Dit gebeurt veelal door medewerkers van ICT- of EPD-afdelingen van zorginstellingen. Het bestand wordt verstuurd naar databewerker Medical Research Data Management (MRDM), waar de data wordt bewerkt, versleuteld en opgeslagen. In lijn met de huidige privacywetgeving zijn de gegevens niet meer herleidbaar tot de individuele patiënt.

## Huidige stand van zaken

De afgelopen jaren zijn diverse fasen doorlopen om DPARD door te ontwikkelen tot een volwaardige kwaliteitsregistratie. Sinds de initiatiefase is er een stevig fundament gebouwd in de datastructuur van de registratie, de verzameling van data en de terugkoppeling aan ziekenhuizen in online dashboards. Vijf jaar na oprichting zijn er 48.152 patiënten geïncludeerd in DPARD, afkomstig uit 29 medische centra in Nederland (Figuur 1). Het overgrote deel van deze patiënten is onder chronische behandeling en wordt meerdere jaren vervolgd in de tijd. Momenteel zijn 62 medische centra (86% van het totaal) aangemeld voor deelname aan DPARD (Figuur 2), waarvan 60% in de testfase van aanlevering zit of data aanlevert. Een belangrijke stap in het landelijk uitrollen van de registratie is het standpunt dat de Nederlandse Internisten Vereniging (NIV) heeft ingenomen. Tijdens een algemene ledenvergadering eind 2020 is besloten dat alle internisten vanaf 2022 inzicht moeten hebben in kwaliteitsinformatie over de verleende diabeteszorg in hun centrum.^3^ Deze stap heeft gezorgd voor prioritering binnen ziekenhuizen en voor landelijke bekendheid. Het effect hiervan is evident zichtbaar in de inclusiecijfers. Ook de impact van de COVID-19-pandemie is duidelijk zichtbaar door de stagnatie van inclusie van patiënten in 2020-2021.

**Figure 1:**
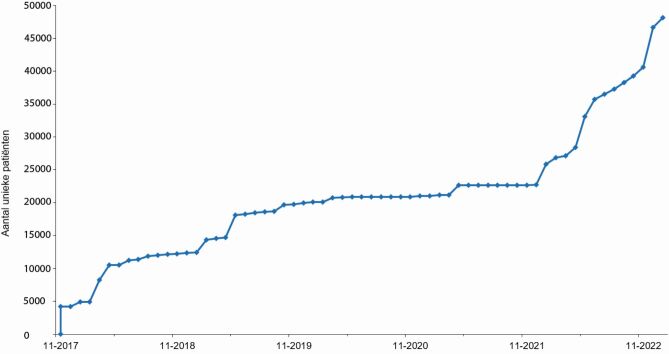

**Figure 2:**
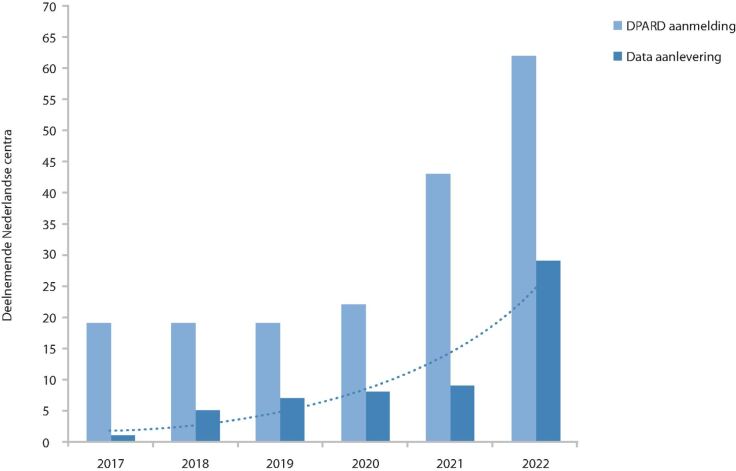

Wat verder heeft bijgedragen aan de opkomst van DPARD is de samenwerking die is opgezocht met de twee grootste EPD-leveranciers van Nederland, om de registratie te integreren in het zorgproces en registratie aan de bron te vergemakkelijken. Uitgangspunt is dat DPARD het zorgproces vastlegt zoals het geregistreerd wordt in het EPD. Daarom wordt binnen het dagelijkse team achter DPARD veel aandacht besteed aan het optimaliseren van de aansluiting op het zorgproces en de verschillende EPD's teneinde registratielast te beperken. Dit is van belang omdat de prevalentie van patiënten met diabetes mellitus in Nederland naar verwachting de komende jaren verder zal blijven stijgen.^4,5^ Tot slot sluit DPARD sinds 2022 volledig aan op de jaarlijks verplicht aan te leveren landelijke kwaliteitsinformatie (diabetesindicatoren), waardoor het mogelijk is om al deze indicatoren eenvoudig via DPARD aan te leveren.

## Toekomstplannen

Het hoofddoel van kwaliteitsregistraties is om het zorgproces inzichtelijk te maken met behulp van kwaliteitsinformatie, deze te evalueren en vervolgens in te zetten voor continue verbetering van de geleverde zorg. Er wordt toegewerkt naar het volledig doorlopen van deze verbetercyclus. Ondanks het recente eerste lustrum is DPARD nog steeds een registratie in ontwikkeling. Het bereiken van landelijke dekking voor DPARD binnen de interne geneeskunde en de kindergeneeskunde is op dit moment een prioriteit, zodat de patiëntkenmerken, uitkomsten en praktijkvariatie tussen ziekenhuizen en regio's van de Nederlandse diabeteszorg inzichtelijk worden. Ook is het essentieel dat de verzamelde gegevens valide en betrouwbaar zijn en blijven. Om deze reden is er een dataverificatietraject gestart, waarbij de inclusie van patiënten en enkele belangrijke klinische variabelen geverifieerd worden in deelnemende centra met behulp van administratieve ziekenhuisdata. Om in de toekomst gedegen uitspraken te kunnen doen over de kwaliteit van zorg, is correctie voor patiëntgebonden karakteristieken ook van belang, de zogeheten casemixcorrectie. Ten slotte wordt in 2023 een start gemaakt met het integreren van *patient reported outcome measures* (PROMs) binnen DPARD, conform de basisset diabetes zoals geformuleerd door de International Consortium for Health Outcomes Measurement (ICHOM).^6^ Om inzicht te krijgen in uitkomsten van zorg en praktijkvariatie zijn de door patiënten gerapporteerde uitkomstmaten van belang om vast te stellen of patiënten ook daadwerkelijk baat hebben bij een behandeling.^7^ Op deze manier kunnen de uitkomsten ook ingezet worden om samen met de patiënt te beslissen in de spreekkamer, en kan DPARD duurzaam bijdragen aan verbetering van de kwaliteit van zorg voor de patiënt met diabetes.

## Dankwoord

DPARD is bij uitstek een *joint effort* van veel partijen. Wij danken de leden van de Clinical Audit Board van DPARD, de Wetenschappelijke Commissie en alle betrokkenen binnen the Dutch Institute for Clinical Auditing (DICA) voor hun inzet. Wij danken de Diabetes Vereniging Nederland, de Nederlandse Internisten Vereniging, de Nederlandse Vereniging van Kindergeneeskunde en de Nederlandse Diabetes Federatie voor hun steun. Tot slot danken wij alle participerende centra voor hun inspanningen.

## REFERENTIES

1. Bak JCG, Mul D, Serné EH, de Valk HW, Sas TCJ, Geelhoed-Duijvestijn PH, et al. DPARD: rationale, design and initial results from the Dutch national diabetes registry. BMC Endocr Disord. 2021 Dec;21(1).
2. Nivel | Kennis voor betere zorg. Nederlands Instituut voor Onderzoek van de Gezondheidszorg [Internet]. Available from: https://nivel.nl/nl
3. de Vries S, Bak J, Verheugt C. Diabeteszorg naar een hoger niveau. Interne Geneeskd Mag voor internist [Internet]. 2021;3(12):18-20. Available from: https://www.internisten.nl/over-de-niv/magazine/
4. Sun H, Saeedi P, Karuranga S, Pinkepank M, Ogurtsova K, Duncan BB, et al. IDF Diabetes Atlas: Global, regional and country-level diabetes prevalence estimates for 2021 and projections for 2045. Diabetes Res Clin Pract. 2022;183:109119.
5. Nielen M, Poos R, Korevaar J. Diabetes mellitus in Nederland. Prevalentie en incidentie: heden, verleden en toekomst. Utrecht; 2020.
6. Nano J, Carinci F, Okunade O, Whittaker S, Walbaum M, Barnard-Kelly K, on behalf of the DWG of the IC for HOM (ICHOM). A standard set of person-centred outcomes for diabetes mellitus results of an international and unified approach. Diabet Med. 2020;37(12):1959-2172.
7. Lamberts MP, Drenth JPH, Van Laarhoven CJHM, Westert GP. Uitkomst van behandeling volgens patiënten: Instrument om variaties in klinisch handelen terug te dringen. Ned Tijdschr Geneeskd. 2013;157(11):1-4.

Highlights

- Landelijke diabetesregistratie DPARD is eind 2017 opgericht en bestaat inmiddels vijf jaar.
- DPARD is een voorbeeld van een multidisciplinaire registratie, waarin poliklinisch behandelde kinderen en volwassenen met diabetes mellitus vervolgd worden over de tijd.
- De patiëntenvereniging en wetenschappelijke verenigingen zijn actief betrokken en onmisbaar in de ontwikkeling van de registratie.
- Inmiddels levert 40% van de Nederlandse centra gegevens aan en is 86% aangemeld.
- Aangeleverde kwaliteitsgegevens zijn inzichtelijk in interactieve dashboards en kunnen vergeleken worden met andere deelnemende instellingen.
- Directe extractie van gegevens uit het EPD helpt om registratielast te beperken.
- Het hergebruiken van data kan verder worden geoptimaliseerd als er aandacht blijft bestaan voor het gestructureerd vastleggen van gegevens in EPD's.

